# From Dizziness and Hearing Loss to Coma: A Case of Basilar Artery Occlusion

**DOI:** 10.7759/cureus.43165

**Published:** 2023-08-08

**Authors:** Tiago Valente, Valter Duarte, Laura Baptista, Joana Melo, Gorete Jesus

**Affiliations:** 1 Internal Medicine, Centro Hospitalar do Baixo Vouga, Aveiro, PRT

**Keywords:** hearing loss, coma, dizziness, posterior circulation stroke, basilar artery occlusion

## Abstract

Basilar artery (BA) occlusion is a rare and devastating cause of ischemic stroke. Presenting symptoms are frequently non-specific and include dizziness, vertigo, nausea, vomiting, headache, and, rarely, hypoacusis. Clinical history and appropriate neurological evaluation are essential for diagnosis. We present the case of a 65-year-old female with dizziness, vomiting, dysarthria, and hearing loss, progressing to right-side hemiparesis and decreased level of consciousness culminating in a coma in just a few hours. She had an atherothrombotic BA occlusion and was submitted to mechanical thrombectomy with full artery recanalization, resulting in rapid neurological improvement in the first days after treatment and almost full recovery during the following months. Early suspicion of posterior circulation stroke from non-specific symptoms is paramount for correct diagnosis and timely treatment, which has an important impact on disability and mortality. Early and complete BA recanalization can result in a positive outcome in a disease that would otherwise be extremely severe. All physicians should be aware of a possible posterior circulation stroke in patients presenting with dizziness, vertigo, vomiting, or sudden hypoacusis and should meticulously search for specific signs or symptoms of neurological dysfunction such as nystagmus, gaze palsies, dysarthria, hemiparesis, or a decreased level of consciousness.

## Introduction

The posterior circulation of the brain includes the paired vertebral arteries (VAs), the basilar artery (BA), the posterior cerebral arteries, and all their branches, supplying the brain stem, cerebellum, thalamus, and occipital cortex [1].

A posterior circulation stroke (PCS) results from the occlusion of any of these arteries, which is mostly caused by in situ thrombosis or thromboembolic disease from an atherosclerotic plaque, cardioembolic disease, or artery dissection [1]. PCSs account for about 20% of all ischemic strokes [2]. BA occlusion (BAO) accounts for 1% of all strokes [2].

Presenting symptoms are frequently non-specific, contributing to the difficulty of diagnosis. These include dizziness, nausea or vomiting, headache, and hearing loss [3]. Vertigo and dizziness are common non-specific symptoms in patients presenting to the emergency department (ED), making it a challenge to distinguish between benign and life-threatening diseases [1]. These symptoms are two of the most prevalent complaints in the ED, affecting 15 to 35% of the general population and with an estimated 7 million clinic visits in the United States each year [4,5]. These symptoms are two of the most prevalent complaints in the emergency department (ED), affecting 15 to 35% of the general population and with an estimated 7 million clinic visits in the United States each year [4,5]. Peripheral vestibular causes, including benign paroxysmal positional vertigo, labyrinthitis, and Meniere's disease, are responsible for almost half of all cases, while cerebrovascular disease accounts for approximately 6% of cases [4].

Depending on the site of obstruction and extent of the ischemic lesion, BAO can cause hemiplegia or quadriplegia, reduced consciousness, dysarthria, dysphagia, oculomotor dysfunction, or other cranial nerve palsies [2].

## Case presentation

The patient was a 65-year-old female, with a modified Rankin Scale (mRS) score of zero, and a prior medical history of arterial hypertension, dyslipidemia, and anxiety, presenting to the ED with a history of headache, dizziness, and vomiting beginning two days before admission. She was described as having a Glasgow Coma Scale (GCS) score of 15 and a normal physical exam except for a blood pressure (BP) of 176/55 mmHg. However, observation was remarkably lacking other medical history details and exclusion of other symptoms, a summary neurological examination, and even evaluation of other vital signs. She was given diazepam 10mg for presumed anxiety. On later reevaluation, BP dropped to 117/70 mmHg, but the patient was showing increased somnolence, motivating the physician to request a head computed tomography (CT) scan, which was normal. Once again, no neurological exam was performed.

**Figure 1:**
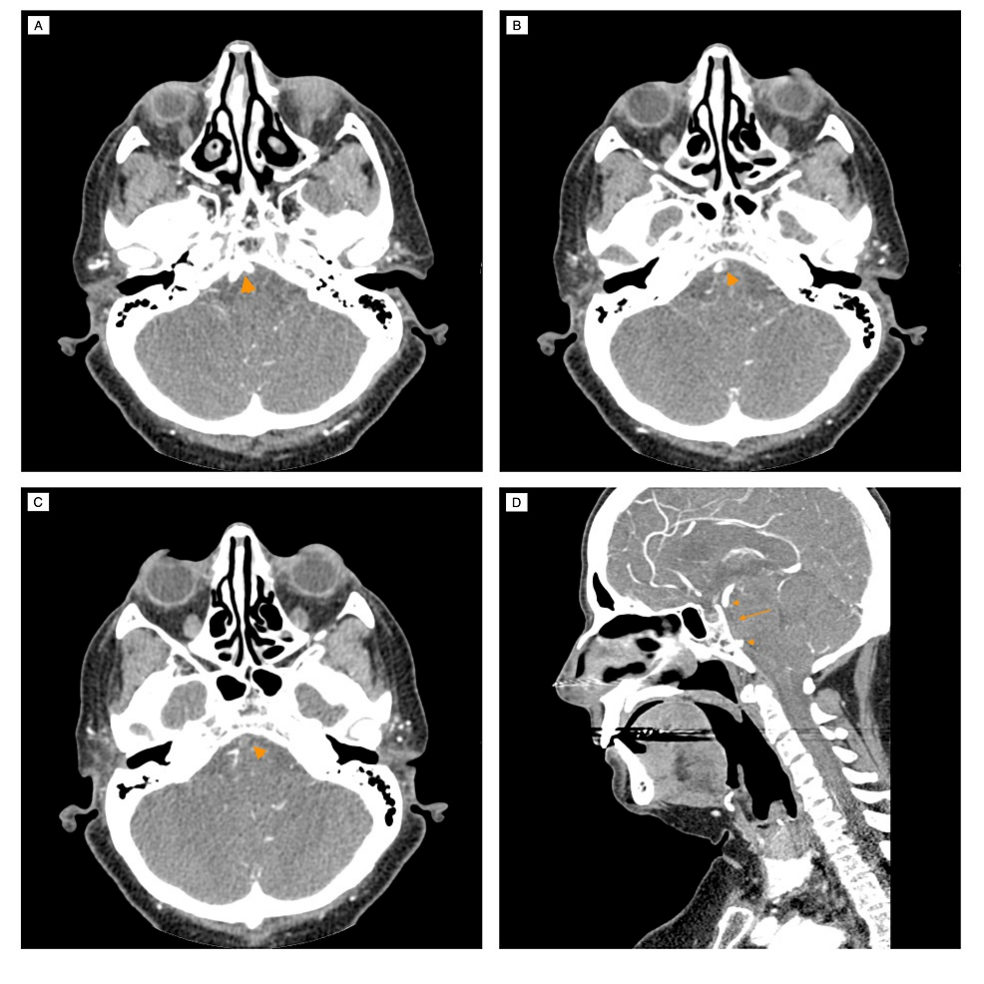
Computed tomography cerebral angiography showing basilar artery occlusion. (A) shows both vertebral arteries joining to form the basilar artery (arrowhead). (B) shows the beginning of the basilar artery with a filling defect caused by an endoluminal thrombus (arrowhead). (C) shows total absence of contrast opacification of the basilar artery (arrowhead). (D) is a reconstructed sagittal section showing the extent of the basilar artery thrombus, occupying its proximal and middle segments (arrow); contrast opacification stops at the beginning of the basilar artery (arrowhead) and is again evident at the distal portion of the basilar artery (arrowhead) because of retrograde flow through the anterior circulation.

Approximately seven hours after admission to the ED, the patient had a decreased level of consciousness and was described as more somnolent, expressing fewer words than before and with slurred speech. Only at this time was the patient’s family contacted, and a more accurate medical history was taken: the patient was having frequent dizziness episodes in the past few weeks and was brought to the ED because she showed new onset vomiting 2 days before and hearing impairment and slightly slurred speech a few hours before hospital admission. At reevaluation, the patient had a GCS score of 14, with fluctuant cooperation during the exam, not responding to simple questions such as age and present month but was still capable of performing simple commands such as closing her eyes or squeezing her left hand. She had a preferential gaze to the left but was able to overcome to the right on command. No visual field defects were detected; she had a normal pupil size and reactivity, and a right-beating nystagmus was observed when overcoming preferential gaze. No facial palsy was apparent. She showed right-sided hemiparesis with right-arm plegia scoring 0/5 and right-leg paresis scoring 2/5 on the Medical Research Council (MRC) scale. She also appeared to have left-sided hemiparesis, scoring 3/5 on the MRC scale on both left limbs. Limb ataxia could not be tested due to a lack of cooperation or understanding. She had no apparent peripheral sensory loss to tactile stimulation. At that time, she couldn’t name objects or repeat simple phrases but speech comprehension was normal as she could perform simple tasks and language function did not seem to be affected as she could be clearly understood when referring to noting hypoacusis while pointing to her left ear. She had mild to moderate dysarthria, with slurred and slow speech but easily understandable. No visual or tactile extinction was noted. The patient scored 15 points on the National Institutes of Health Stroke Scale (NIHSS). A decreased level of consciousness, decreased global strength but predominantly right-sided, preferential left gaze, dysarthria, vomiting, hearing loss, and nystagmus, strongly pointed to a PCS, localized to the pons and cerebellum. She was submitted to an emergent CT angiography, which revealed BAO by an endoluminal thrombus on the proximal and middle portions of the BA (Figure 1).

**Figure 2:**
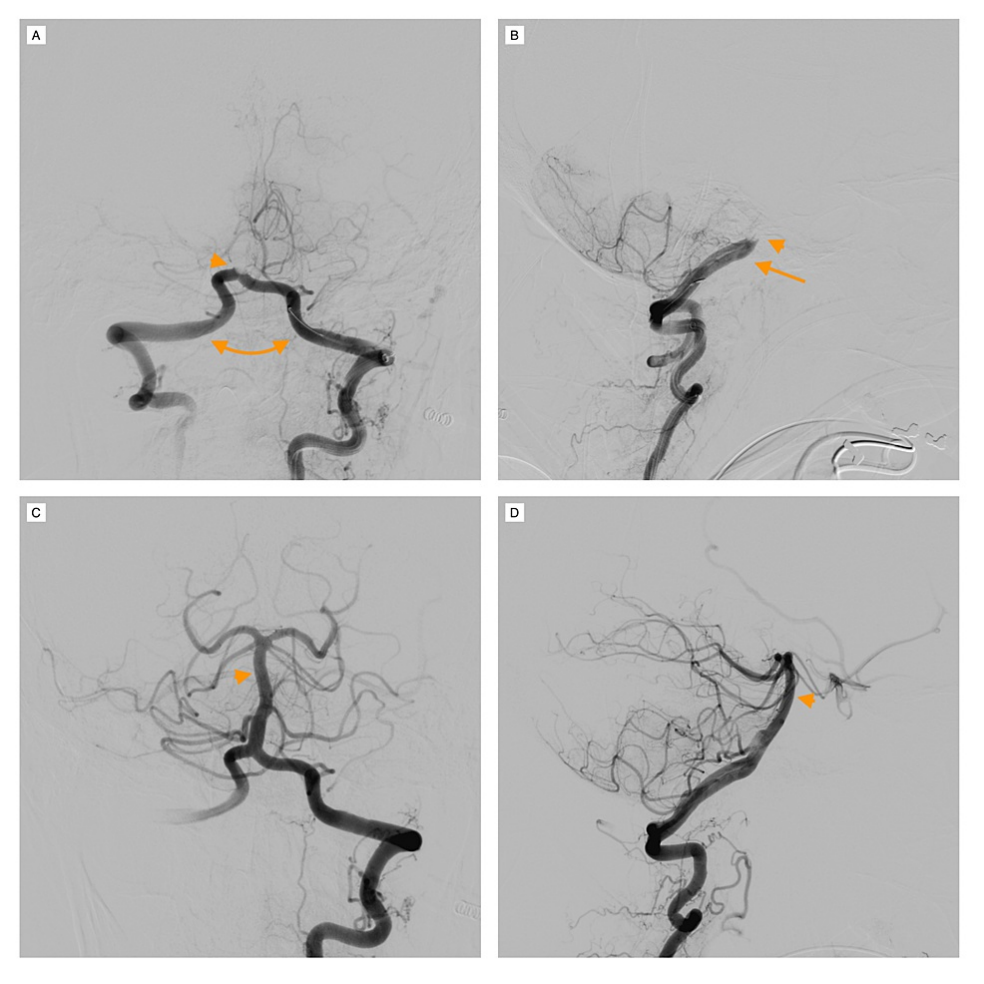
Conventional angiography and mechanical thrombectomy resulting in total repermeabilization. Anteroposterior (A) and lateral (B) projections show both vertebral arteries (arrows) joining to form the basilar artery but with no contrast filling it, confirming basilar artery occlusion right at the vertebrobasilar junction (arrowhead). (C) and (D) are anteroposterior and lateral projections (respectively) of the vertebrobasilar circulation after repermeabilization, showing full opacification of the basilar artery by contrast (arrow), as well as the paired posterior cerebral arteries (dotted arrows), superior cerebellar arteries (upper arrowheads), and anterior inferior cerebellar arteries (lower arrowheads).

In the meantime, the patient’s level of consciousness decreased further, requiring urgent orotracheal intubation for airway protection. She was submitted to conventional angiography, confirming proximal BAO right at the vertebrobasilar junction and mechanical thrombectomy was performed, resulting in total artery repermeabilization (thrombolysis in cerebral infarction [TICI] grade 3, see Figure 2).

**Figure 3:**
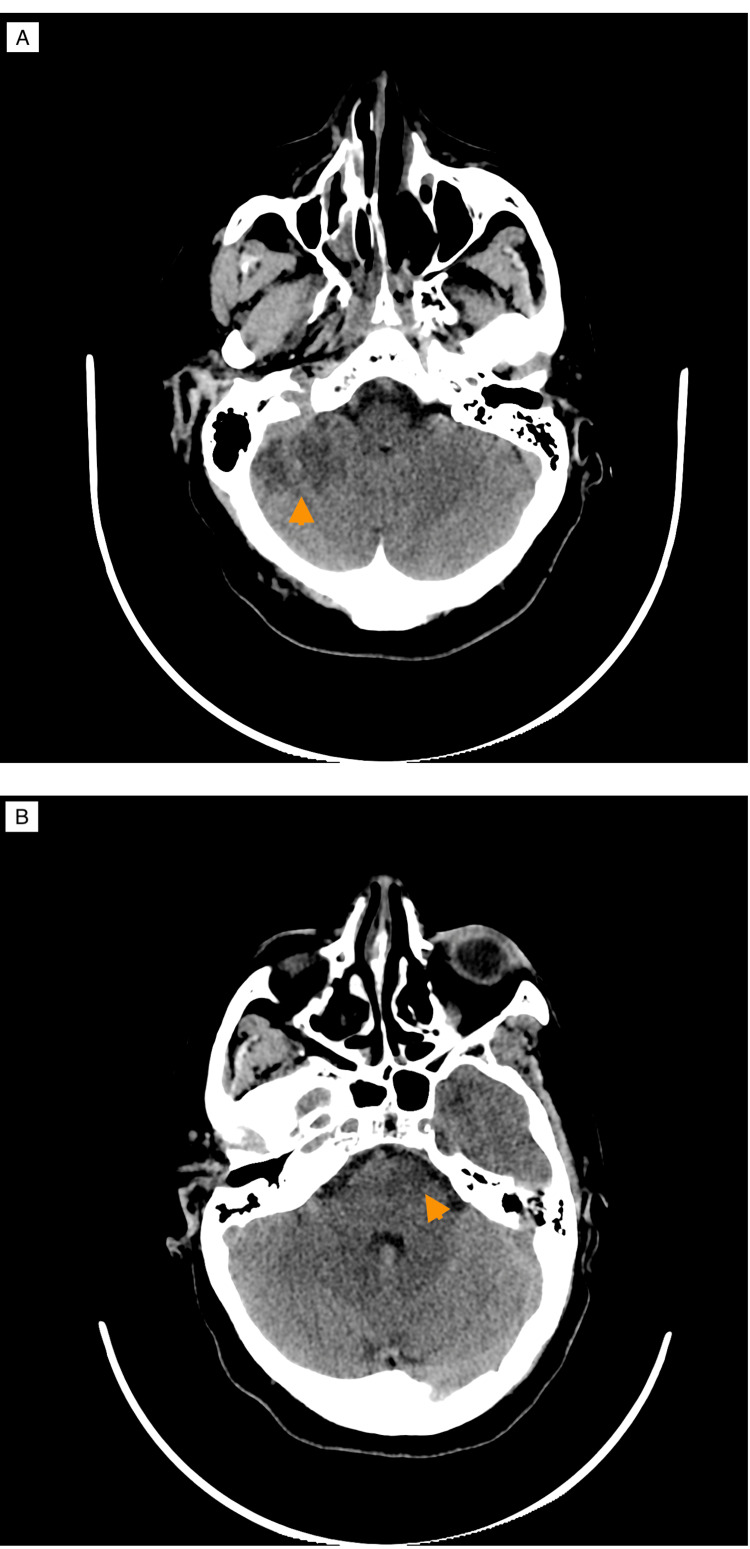
Head computed tomography scan depicting resultant ischemic lesions from basilar artery occlusion. (A) shows a hypodense right cerebellar lesion corresponding to the territory supplied by the right anterior inferior cerebellar artery (arrowhead). (B) shows a hypodense left pontine lesion corresponding to the territory supplied by the basilar artery.

A control head CT scan was made, showing a hypodense right cerebellar lesion in the territory supplied by the Anterior Inferior Cerebellar Artery (AICA) and a hypodense left pontine lesion (Figure 3).

The patient’s condition improved in the following days, with progressive improvement of the level of consciousness and neurological deficits. Thirteen days after endovascular treatment, she had slight dysarthria, right lower facial palsy, and right-side hemiparesis, scoring 3/5 on the MRC scale. After six months of rehabilitation, the patient had almost fully recovered, scoring one on the mRS.

## Discussion

Diagnosing a posterior circulation ischemic stroke can be challenging. Although BA occlusion is a rare cause of stroke, accounting for 1% of all strokes, it carries a poor prognosis and a high risk of severe disability and death if not readily identified and treated [2]. In the New England Medical Center Posterior Circulation Registry, the most frequent symptoms of vertebrobasilar ischemia were dizziness, nausea or vomiting, headache, unilateral limb weakness, and dysarthria, but other less frequent symptoms included diplopia, limb sensory deficit, bilateral limb weakness, and hearing loss [3]. Audiovestibular loss is an important sign of stroke affecting AICA territory [6]. The internal auditory (or labyrinthine) artery is solely responsible for supplying the internal ear [6] and usually arises from the AICA, but in some cases arises directly from the BA [2,7].

Atherosclerotic disease often involves the VAs bilaterally and the proximal and middle segments of the BA. Prodromal symptoms and a progressive disease course are suggestive of atherosclerotic disease, most often with proximal lesions. Up to two-thirds of patients with BAO have prodromal transient ischemic attacks (TIAs) [2]. Vertigo and headache are the most frequent prodromal symptoms, but less common symptoms include a decreased level of consciousness, diplopia, visual field deficits, hemiparesis, hemihypesthesia, disequilibrium, dysarthria, facial palsy, tinnitus, and auditory loss. Occlusion of the proximal and middle segments of the BA usually causes pontine ischemia resulting in hemi or quadriplegia, reduced consciousness, dysarthria, horizontal gaze palsy, and other cranial nerve deficits.

This case shows the basilar importance of good clinical history taking and a detailed physical examination even in patients with mild and seemingly benign symptoms. The patient exhibited dizziness episodes weeks before the acute stroke, which were probable TIAs heralding atherosclerotic obstruction of the vertebrobasilar circulation. As the obstruction progressed and the BA became acutely occluded by a thrombus, new symptoms such as vomiting, dysarthria, and hearing loss ensued, steadily progressing to quadriparesis and coma.

Although time is short for an evaluation in the emergency department, missing a stroke diagnosis means delaying effective treatment and compromising the patient’s recovery or even his/her life. Early partial or complete recanalization of the occluded artery has a positive impact on recovery [7]. Although the patient recovered well, correct clinical history taking and an appropriate physical and neurological examination could have permitted an earlier vertebrobasilar ischemic stroke diagnosis and its treatment.

## Conclusions

PCS can present with non-specific symptoms, such as dizziness, headache, vomiting, and hearing loss, which are common to other more frequent and benign diseases, warranting the need for a thorough neurological exam in patients manifesting these symptoms. Efforts need to be employed to exclude focal neurological signs, such as other cranial nerve deficits or hemiparesis.

It is extremely important to identify BAO early in the course of the disease in order to administer effective treatment and improve outcomes. A delayed or missed diagnosis of posterior circulation stroke can have dire consequences, including severe disability and death that could have been otherwise prevented with appropriate acute stroke treatment.
